# BioNetGMMFit: estimating parameters of a BioNetGen model from time-stamped snapshots of single cells

**DOI:** 10.1038/s41540-023-00299-0

**Published:** 2023-09-22

**Authors:** John Wu, William C. L. Stewart, Ciriyam Jayaprakash, Jayajit Das

**Affiliations:** 1 Department of Computer Science, The Ohio State University, 281 W Lane Ave, Columbus, OH 43210 USA; 2 Steve and Cindy Rasmussen Institute for Genomics, The Abigail Wexner Research Institute, Nationwide Children’s Hospital, 700 Children’s Drive, Columbus, OH 43205 USA; 3 GIG Statistical Consulting LLC, Columbus, OH 43210 USA; 4 Department of Physics, The Ohio State University, 191 W Woodruff Ave, Columbus, OH 43210 USA; 5 Departments of Pediatrics, Biomedical Informatics, Pelotonia Institute of Immuno-Oncology, College of Medicine, and Biophysics Program, The Ohio State University, 370 W 9th Ave, Columbus, OH 43210 USA

**Keywords:** Software, Time series

## Abstract

Mechanistic models are commonly employed to describe signaling and gene regulatory kinetics in single cells and cell populations. Recent advances in single-cell technologies have produced multidimensional datasets where snapshots of copy numbers (or abundances) of a large number of proteins and mRNA are measured across time in single cells. The availability of such datasets presents an attractive scenario where mechanistic models are validated against experiments, and estimated model parameters enable quantitative predictions of signaling or gene regulatory kinetics. To empower the systems biology community to easily estimate parameters accurately from multidimensional single-cell data, we have merged a widely used rule-based modeling software package BioNetGen, which provides a user-friendly way to code for mechanistic models describing biochemical reactions, and the recently introduced CyGMM, that uses cell-to-cell differences to improve parameter estimation for such networks, into a single software package: BioNetGMMFit. BioNetGMMFit provides parameter estimates of the model, supplied by the user in the BioNetGen markup language (BNGL), which yield the best fit for the observed single-cell, time-stamped data of cellular components. Furthermore, for more precise estimates, our software generates confidence intervals around each model parameter. BioNetGMMFit is capable of fitting datasets of increasing cell population sizes for any mechanistic model specified in the BioNetGen markup language. By streamlining the process of developing mechanistic models for large single-cell datasets, BioNetGMMFit provides an easily-accessible modeling framework designed for scale and the broader biochemical signaling community.

## Introduction

Recent advancements in single-cell technologies have allowed for the measurement of cell-to-cell differences in mRNA/protein abundances^1–3^. These differences enable the evaluation of means and higher-order moments (e.g., variances and covariances), which can be utilized to improve the estimation of model parameters. One potential strategy, which is especially useful in our case where the probability distribution for the data (i.e., the likelihood) is unknown, is to estimate model parameters by minimizing the differences between sample moments and their corresponding predicted moments computed from in silico models. However, it is not obvious how any set of moment differences should be summarized, especially since sample moments (and hence moment differences) can vary considerably in single-cell data. The Generalized Method of Moments (GMM), widely used in econometrics^4,5^ and described in greater detail below, provides a systematic approach to incorporate means and higher-order moments in parameter estimation^6,7^. Within GMM, the moment differences are combined into a single measure of cost (i.e., distance) using a system of weights that efficiently accounts for fluctuations across sample moments (see A.1 GMM Primer for more details). In practice, to find parameter values that minimize the GMM cost, one usually needs an optimization algorithm such as gradient-descent^8^ or stochastic algorithms, such as simulated annealing^9^ or parallel tempering^10,11^. In recent years, a class of meta-heuristic optimization algorithms, such as Particle Swarm Optimization (PSO) that do not require the calculation of gradients and can be easily parallelized has been developed (see refs. ^12,13^ for a pedagogical review).

Rule-based modeling approaches, such as BioNetGen^14^ and libRoadRunner^15^, have been developed to address combinatorial complexity in modeling biochemical reactions in signaling and gene regulatory networks^16,17^. These approaches provide a user-friendly way to construct models. Estimating model parameters, such as reaction rates, is crucial for improving model predictions and quantifying underlying mechanisms described by the model using experimental measurements. With the emergence of software packages such as PyBioNetFit^18^, parameter estimation from rule-based models using bulk measurements of selected proteins (e.g., average or total protein abundances observed over time) has become possible.

We introduce BioNetGMMFit, a software tool that uses GMM to improve parameter estimation in BioNetGen models by exploiting the additional information in single-cell snapshot data. This tool requires users to supply a BioNetGen model .bngl file, time-stamped snapshot abundance data files, and run configuration files. BioNetGMMFit utilizes the GMM analysis of time-stamped protein abundances, as implemented in CyGMM^7^, to estimate the parameters of the BioNetGen model, provide confidence intervals, predict moments at future times, and report the minimum cost (i.e., the distance between the sample moments and the moments predicted by the model using the GMM estimate of the parameters). While GMM can be used in conjunction with a wide variety of optimization routines, we use PSO to optimize our cost function because it does not require gradient calculations, scales well with higher-dimensional search spaces, has a relatively short run time, and is easily parallelized on a compute cluster. In addition, users can tune the PSO hyperparameters, which can affect the optimization’s efficiency and accuracy. As such, in addition to being written in C++, BioNetGMMFit is scalable with increasing data sizes on high-performance computing clusters. BioNetGMMFit is also available through Docker as C++ compilation varies across different operating systems. However, for improved performance, BioNetGMMFit can be compiled as a C++ executable through cmake and has been tested on Linux operating systems.

The manuscript is organized as follows. In the Software Description section, we provide detailed information on the implementation and use of BioNetGMMFit. Next, in the “Results section”, we present three examples of modeling single-cell data where we apply BioNetGMMFit and compare it with existing software tools. Finally, we offer our conclusions, and discuss future directions and limitations in “Discussion”.

## Results

**Fig. 1:**
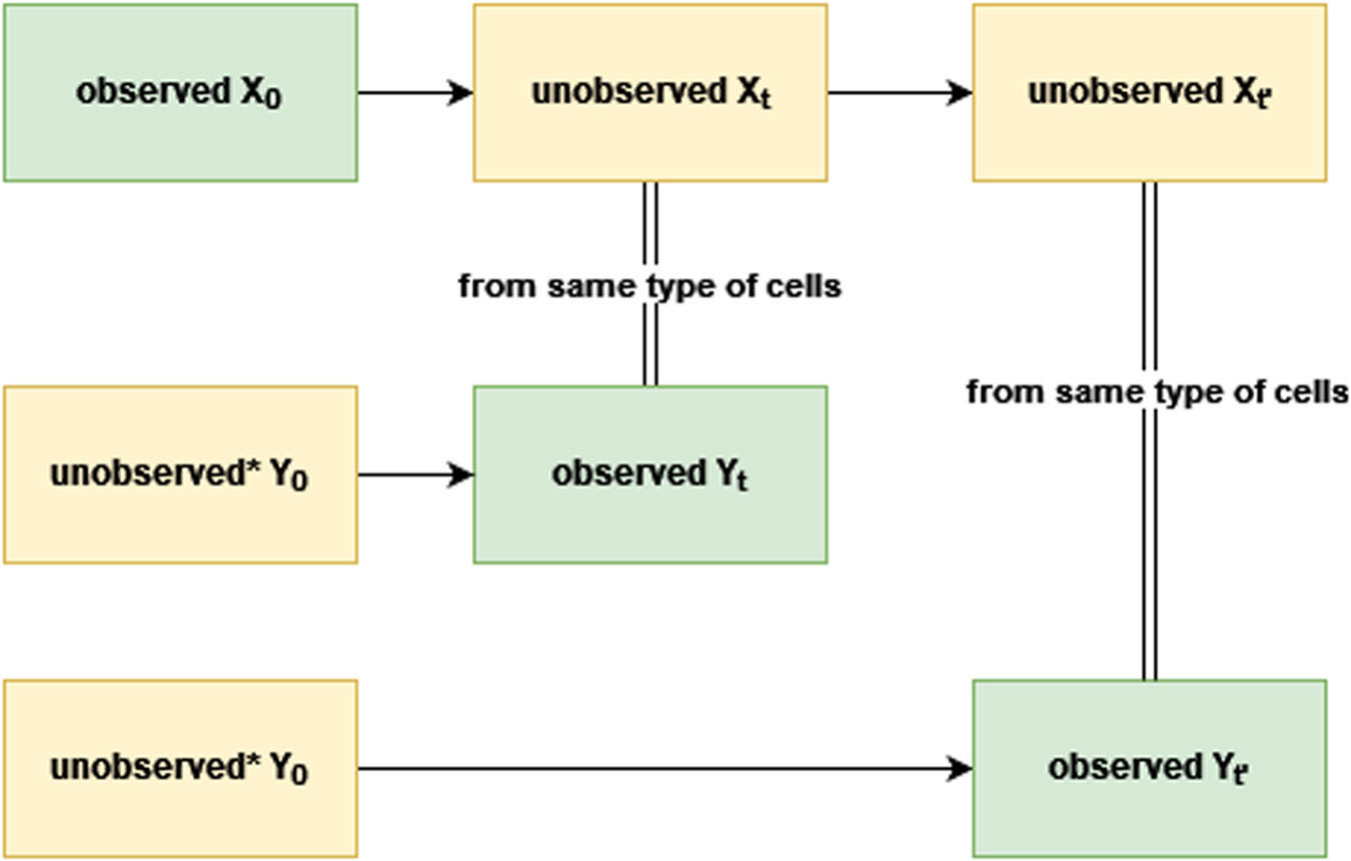
Data generation process. A fundamental constraint of time-stamped CyTOF data is that in order to measure a cell, it must be sacrificed. Therefore, in physical experiments, the initial conditions of cells observed at time *t* and ${t}^{{\prime} }$ are unknown. Similarly, cells observed at time *t*_0_, cannot be observed at future times *t* and ${t}^{{\prime} }$. Here, we refer to such initial conditions and future states (shown in yellow) as “unavailable”. By contrast, parameter estimation only makes use of the “observed” data shown in green. Note that the “unavailable” data marked with an asterisk (*) must be generated in order to simulate data obtained from CyTOF experiments. In particular, one must simulate the initial conditions of all cells so that disjoint subsets of cells can be evolved to future times of interest. For ease of exposition, the subset index is not shown. We use the X and Y labels here in order to remain consistent with the inputs defined in Fig. 10.

We applied BioNetGMMFit to simulated datasets generated by models with known ground truth parameters to provide a reference point to evaluate its robustness, efficacy, and versatility. Specifically, we apply BioNetGMMFit to three simulated datasets, where each dataset is generated from a different model. Furthermore, we showcase the software’s functionality, which includes (1) facilitating estimation of model parameters from different combinations of moments for any rule-based model, (2) fixing subsets of model parameters both for simulation and estimation, and (3) forecasting moments at future time points. For each simulated dataset, we generated two sets of initial conditions from lognormal distributions as they have been observed across a variety of single-cell systems^19,20^. To mimic the experimental constraint that “X” cannot be used to generate the observed snapshot data “Y” at time *t*, we generate another set of unobserved initial conditions that are used to obtain “Y” by evolving the BioNetGen models with ground truth parameters. This feature of simulating data with BioNetGen models when given a set of initial conditions is built into BioNetGMMFit. Figure 1 provides a diagram of this process.

For more information on the simulated datasets themselves, please see the GitHub page (https://github.com/jhnwu3/BioNetGMMFit/tree/main/example) in their respective “X” and “Y” directories.

### A biochemical reaction system with first-order reactions

**Fig. 2:**
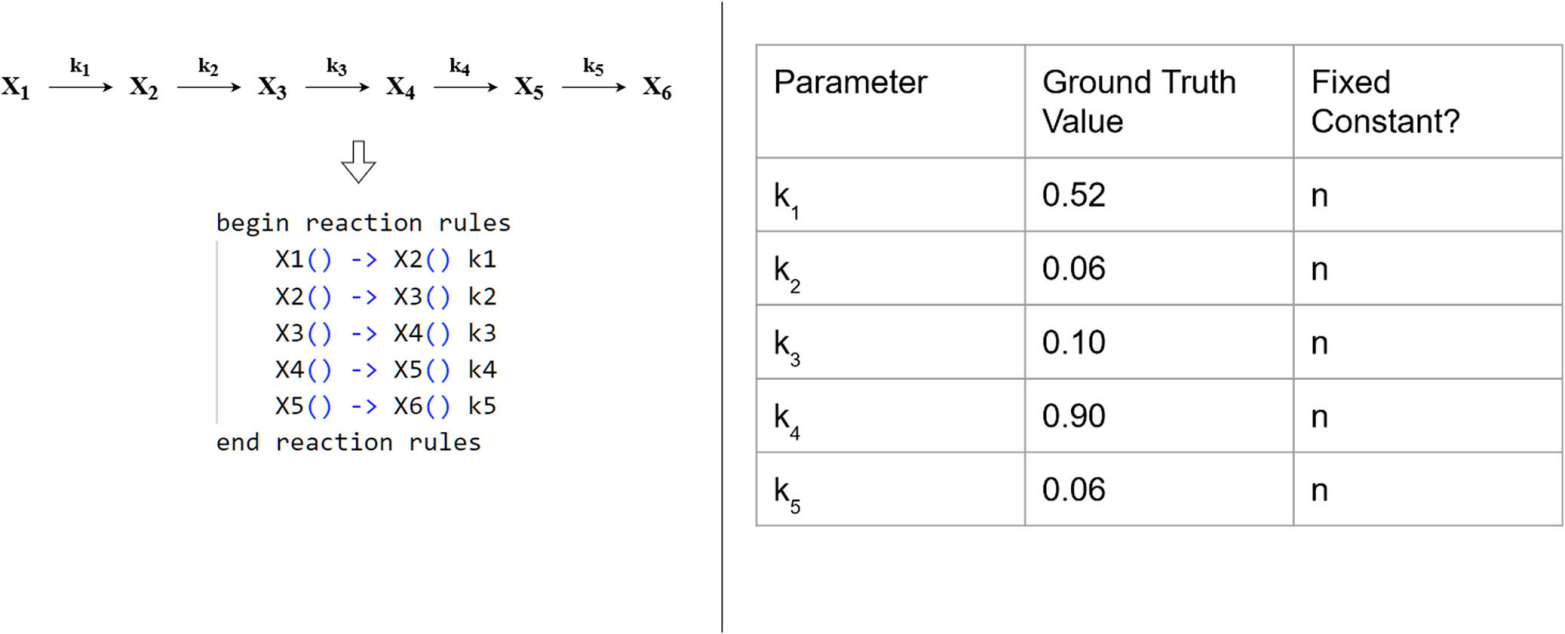
First-order reaction model. On the left, X_1_ through X_6_ represent protein species in a system of reactions where $\mathop{\longrightarrow }\limits^{\theta }$ represents a reaction in which species X_*i*_ is changed into Click to show the PDF X_*j*_ at the rate given by the corresponding *θ*. BioNetGMMFit is used to estimate the set of *θ*’s. Below the system of reactions, its BioNetGen reaction rules are displayed; these need to be defined and input to BioNetGMMFit to estimate the *θ*’s. Note that we use k’s instead of *θ*’s to define model parameters in BNGL. The table on the right contains the ground truth values.

**Fig. 3:**
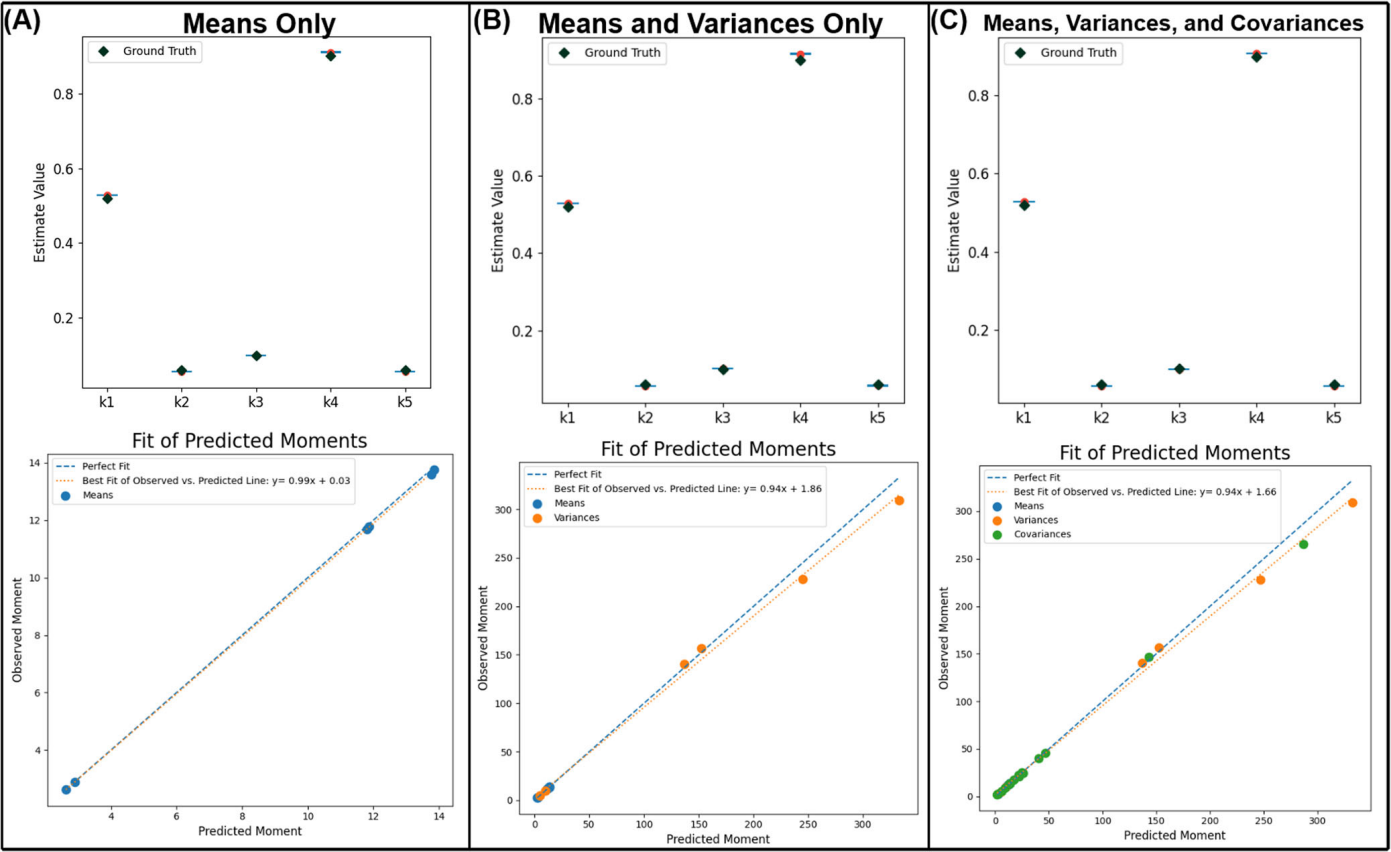
Parameter estimates using different combinations of moments in the first-order reaction model. The left column (**A**) contains 95% confidence intervals of parameter estimates obtained using only the means of the snapshot data at time *t* = 1.5 (top panel) and the comparison of the predicted means and the actual (observed) means (bottom panel). The middle column (**B**) contains analogous results by fitting the means and variances. Note the change in the scale of the values of the moments when the variance is included. The last column (**C**) on the right are the results obtained by fitting all the first, second, and mixed moments or more specifically all 6 means, 6 variances, and 15 covariances of the 6 proteins. This illustrates the flexibility available to the user to choose different subsets of moments for parameter estimation. In the analysis, the initial conditions and the simulated observed conditions at time *t* = 1.5 both contained 10,000 cells each.

Here, we investigate and show that BioNetGMMFit can successfully reproduce the ground truth model parameters for a system of first-order reactions model where six molecular species are arranged in a linear architecture and react via first-order biochemical reactions as illustrated by the reaction network in Fig. 2. The reaction system can represent or can be generalized to represent a variety of sequential cell signaling processes such as phosphorylation of adaptor proteins such as DAP12 or Fc*ϵ*R1*γ* in NK cells^21^ which are phosphorylated by Src family kinases or series of chemical modifications in signaling proteins in membrane-proximal signaling events in T cells^22^. We successfully estimate all 6 parameters of the model and showcase one of BioNetGMMFit’s important features: the ability to estimate parameters using different moments. We note that one advantage of GMM (see Supplementary Material) is that it allows for the use of any number of moments in its objective function. BioNetGMMFit allows the choice of fitting three different combinations of moments of across a dataset: (1) first moments (means), (2) first (means) and second moments (variances), as well as (3) first (means), second (variances), and mixed moments (covariances) as shown in Fig. 3.

A key strength of this flexibility of moment choice in the cost function is the ability to adapt to different problem requirements. For instance, inclusion of higher-order moments tends to improve parameter estimation in this first-order reaction model as shown in Fig. 3, whereas the usage of variances and covariances in its fitting routine produces the tightest confidence intervals around its parameter estimates.

### Model for Msn2-induced transcription in yeast

**Fig. 4:**
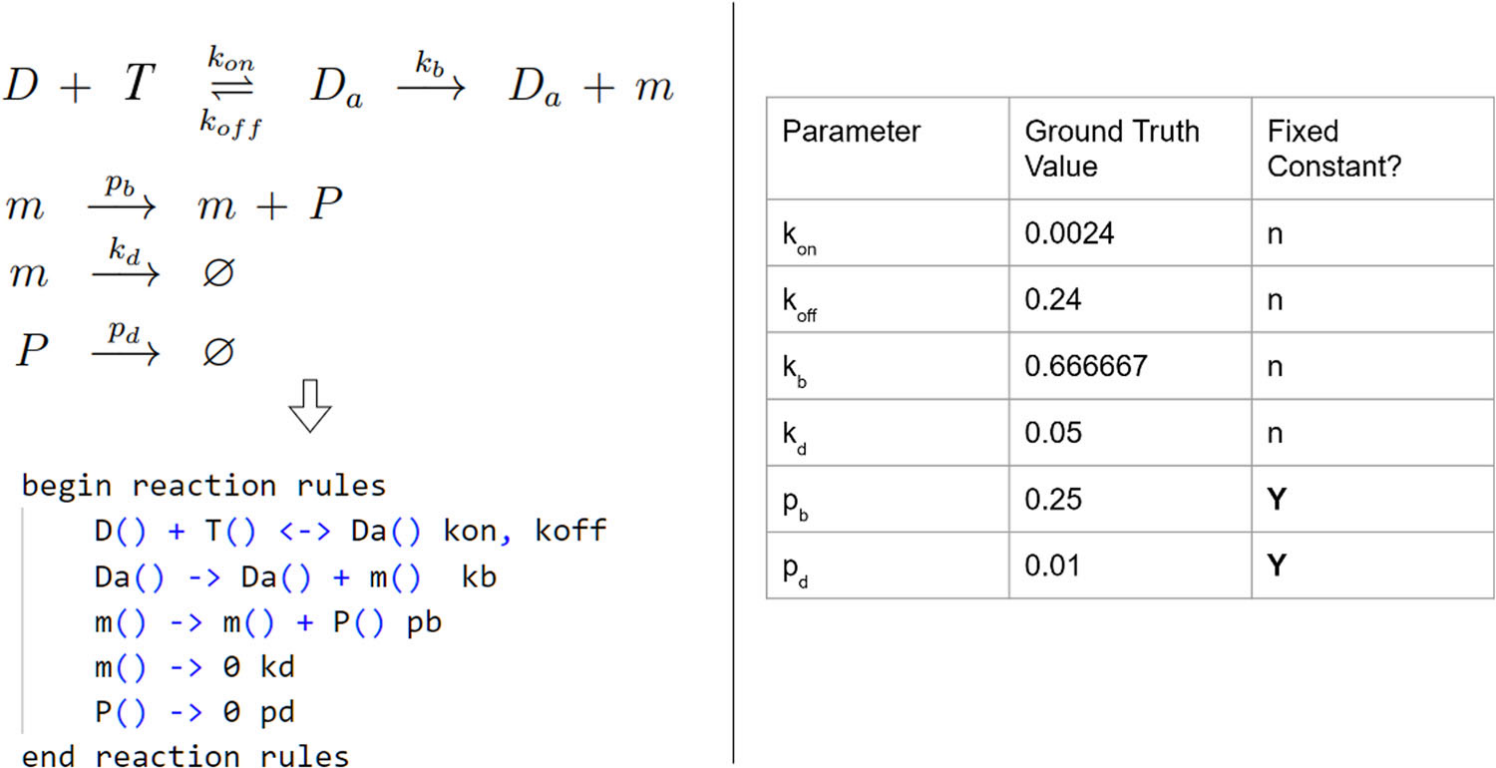
Msn2 transcription model. Model reaction network is shown on the left with its corresponding BioNetGen reaction rules defined below it. Ground truth model parameters and which ones have been fixed are shown in the table on the right. In this case, non-fixed model parameters labeled by “n” are estimated.

Next, as mechanistic models often incorporate some form of nonlinearity, we show that we can successfully perform parameter estimation for a nonlinear gene regulatory model involved in stress response in yeast^23^. This is a simplified version of a commonly occurring gene regulatory motif in yeast. One of the key regulators of the stress response is the transcription factor Msn2 which resides in the cytoplasm in the resting state and is dephosphorylated in response to stress. Upon phosphorylation, Msn2 translocates to the nucleus, binds to stress-responsive elements (STRE), and induces gene expression of response proteins. The model is illustrated in Fig. 4. This example explores a scenario where certain model parameters need to be fixed. Here we fixed two parameters, the death rate *p*_*d*_ and the birth rate *p*_*b*_ of the protein species *P*, reducing the total number of parameters to be estimated to four. The BioNetGen rules describing the model and the ground truth parameter values used to generate the synthetic data are shown in Fig. 4. We analyzed its simulated observed snapshot data at time points 0, 1, and 5 min in its parameter estimation.

**Fig. 5:**
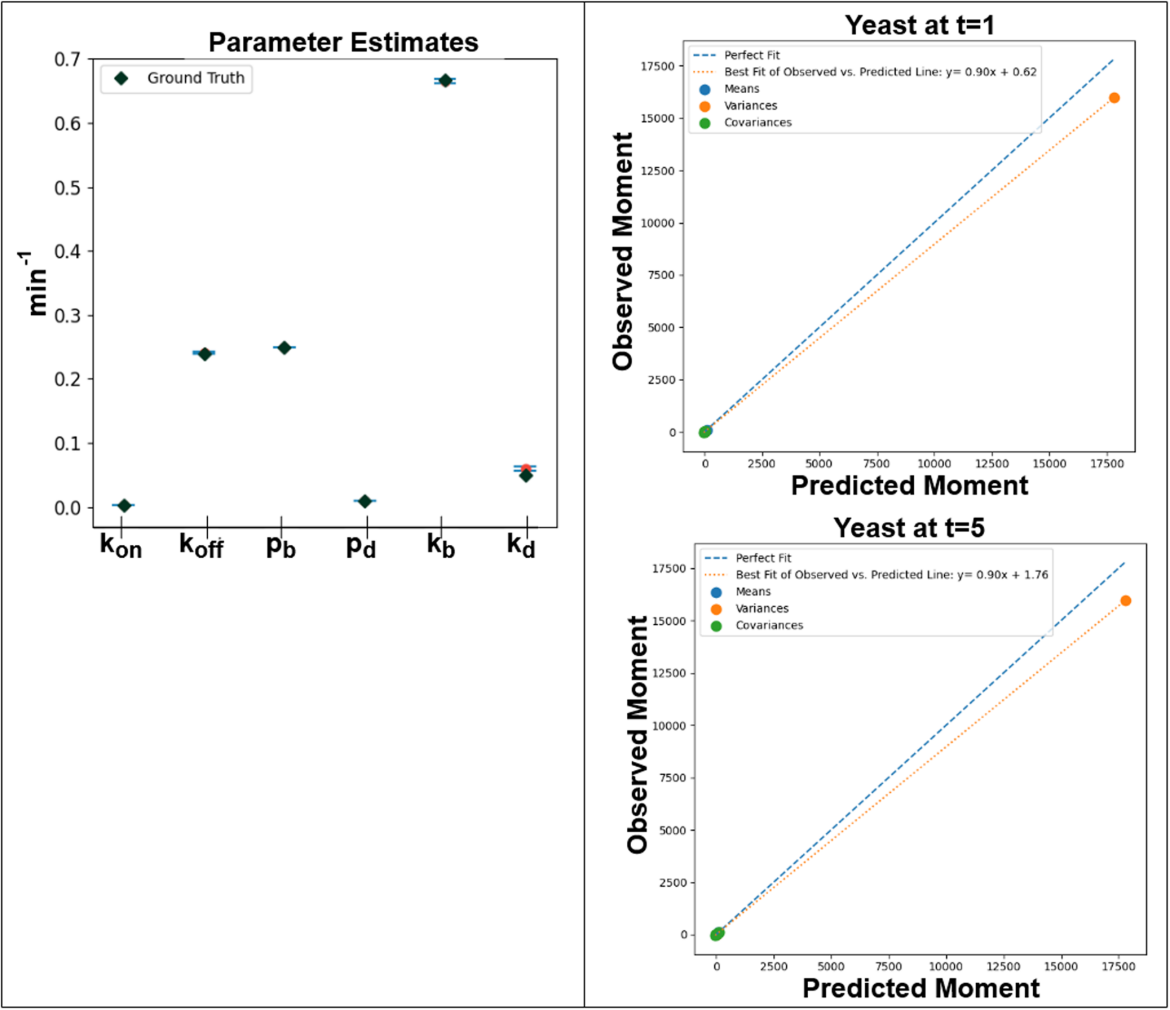
Msn2 transcription model outputs. Model parameter estimate with confidence intervals on the left were generated by the moment fits for two different simulated observed time points, *t* = 1 and *t* = 5 min, on the right. In this case, 95% confidence intervals of parameter estimates shown on the left include the ground truth parameter values. Please note that the middle two x-tick marks, pb and pd are not estimated and are held constant. Using simulated data for the 5 species (i.e D, T_2_, m, etc.) in the ground truth model, 5 means, 5 variances, and 10 covariances across two time points were obtained and used for parameter estimation. All snapshot data used in the analysis contained 10,000 cells.

The parameter estimation results for the Msn2 transcription model are presented in Fig. 5. Although the moment fits for both time points are not perfect, with an underfitted variance for one of the species, the confidence intervals around the estimated parameters fully encompass the ground truth parameters, indicating that BioNetGMMFit is capable of handling parameter estimation for nonlinear models.

### Vav1 activation kinetics in NK cells

**Fig. 6:**
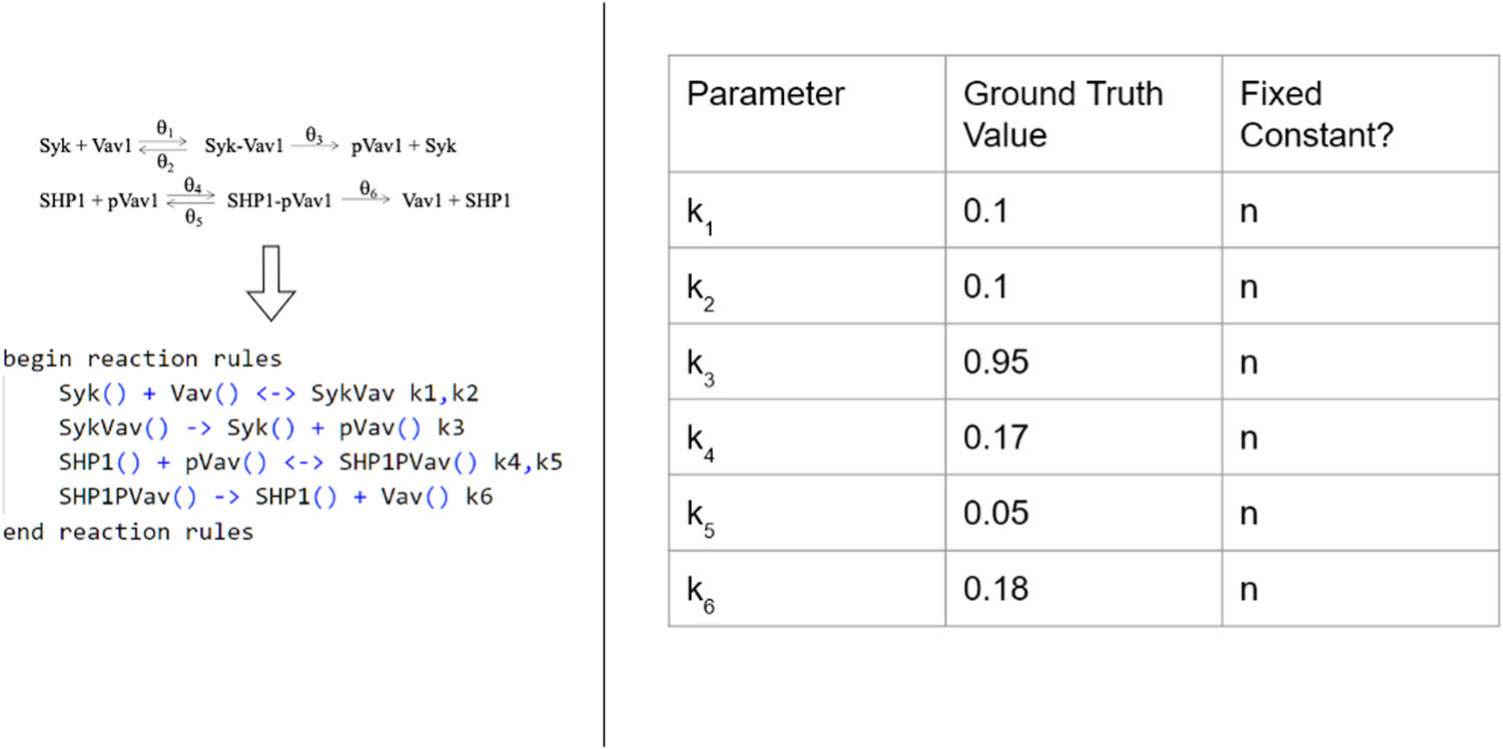
Vav1 activation model. The reaction network displayed on the left represents a simplified version of the phosphorylation-dephosphorylation kinetics of Vav1 in mouse NK cells. The corresponding reaction rules in BioNetGen are shown below. All the parameters, the set of rate constants {*θ*_*j*_} or k_*j*_ in the .bngl file and their ground truth values on the right are estimated using BioNetGMMFit.

As the final example, we consider a simplified model early time biochemical signaling kinetics (Fig. 6) in mouse Natural Killer (NK) cells^24^ to illustrate a case where identifiability issues prevent BioNetGMMFit from capturing the ground truth model parameters. The model describes the phosphorylation of a key signaling protein Vav1 by the kinase Syk bound to activating receptor–ligand complex and the dephosphorylation of phosphorylated Vav1 by the phosphatase SHP1 bound to the inhibitory receptor–ligand complex. The nonlinear biochemical reactions in the model are akin to the zero-order ultrasensitivity model proposed by Goldbeter and Koshland^25^. We assume the abundances for the proteins Syk, Vav1, Syk-Vav1, SHP1, SHP1-pVav1, and pVav1 are measured at any time *t*.

**Fig. 7:**
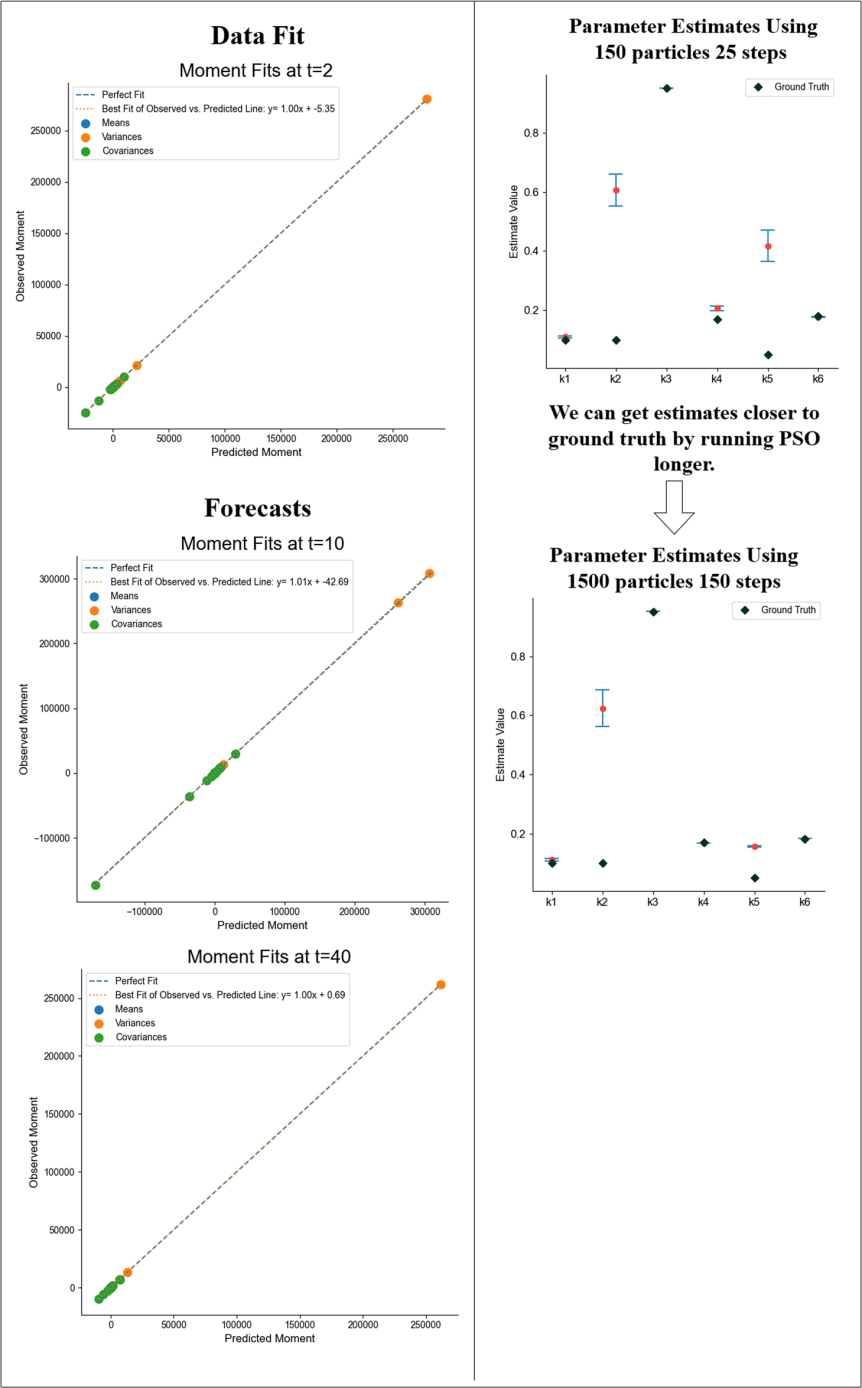
Results for the Vav1 activation model. The ground truth parameter values shown in Fig. 6 are estimated by BioNetGMMFit using data from the ground truth model at *t* = 0.5 and *t* = 2. In total, 5000 cells at each time point were used in the analysis. The panels in the left column show the predicted moments calculated with estimated parameters plotted against the observed moments calculated using the ground truth model at various time points. The top figure shows the comparison at one of the input time points while the bottom two graphs are the “forecast” moments at *t* = 10 and *t* = 40. This shows that such parameter estimates can predict values at time points well into the future. The parameter estimates were derived from configuration 6 Protein A. Please see Supplementary Figure 11 in the Supplementary Material for more information on the PSO hyperparameter configurations used. On the right, we show improvements in estimates by tuning PSO hyperparameters and show parameter estimates for two different hyperparameter configurations. All the moment comparison plots in the left column were generated with parameter estimated obtained using PSO with 1500 particles and 150 steps. Note that the time units for this simulated dataset are arbitrary since BioNetGMMFit does not have units built into its plotting function. However, phosphorylation reactions as defined by the network in Fig. 6 occur on the time scale of seconds.

Following parameter estimation, we predict first, second, and mixed moments. The comparisons between the predictions and the simulated observed moments at different time points are shown in Fig. 7. The three slopes shown in Fig. 7, each near unity, indicate good agreement between the predicted moments (i.e., means, variances, and covariances) and the observed moments at each time point. Furthermore, using the model parameters estimated by BioNetGMMFit, we can *forecast* moments (i.e., predict moments) at times well outside of the given observational range (Fig. 7). The nonlinear Vav1 activation model also presents a case where hyperparameter tuning is needed to generate more accurate parameter estimates. For instance, in Fig. 7, increasing the number of particles and steps in the PSO routine improves the parameter estimates (i.e., brought them closer to the simulated ground truth). Despite improved estimates, this scenario illustrates a parameter estimation problem where other parameter estimates exist that minimize the objective function. Figure 7 shows that although the moment fits are excellent, and the predictions closely match the observed and future unobserved data, the parameter estimates for k_2_ and k_5_ are unable to capture the ground truth values, even with a tuned hyperparameter configuration of 1500 particles and 150 steps of PSO. In the subsection below, we provide further insights into the challenges of parameter estimation in rule-based models.

### Practical concerns in estimation

As seen from the Vav1 activation model, cost functions for ODE models often possess multiple local minima and/or flat regions with small curvatures^7^, that typically make estimation (i.e., the task of finding the best parameter values) challenging for any method. Although PSO mitigates these optimization problems to some extent, depending on the computational constraints, BioNetGMMFit does not fully address the difficulties of such irregular cost functions, e.g., BioNetGMMFit can still yield estimates that reflect a local, rather than the global, minimum. One such example is shown in Fig. 7 where only 150 particles and 25 steps were used for parameter estimation of the simulated Vav1 activation model. We intentionally chose a limited number of particles and steps to examine what could happen in a computationally constrained environment. As one might expect, the estimates for some paremeters were not close to their corresponding ground truth values; however (perhaps surprisingly), the estimates were able to accurately predict the observed first, second, and cross moments at later times as shown in Fig. 7. However, when the number of particles and steps used are increased tenfold, parameter estimates are much closer to the ground truth, although prediction improves only slightly. Moreover, this dramatic improvement in estimation and a slight improvement in prediction demand higher computational costs as shown in Supplementary Fig. 12 where there is a drastic increase in run time from configuration “6 Protein Time Points Set A” to “6 Protein Time Points Set A Extreme” in Supplementary Fig. 12. As such, this raises an interesting question of “How much should one care about obtaining the best possible parameter estimates (as shown with *k*_4_ in Fig. 7 for example), especially when computational resources may be limited and/or when the primary interest of the analysis may be prediction, not estimation?” Furthermore, despite the potential for dramatic improvement in estimation, there is no guarantee that one will realize the desired improvement simply by using more particles and/or steps.

**Fig. 8:**
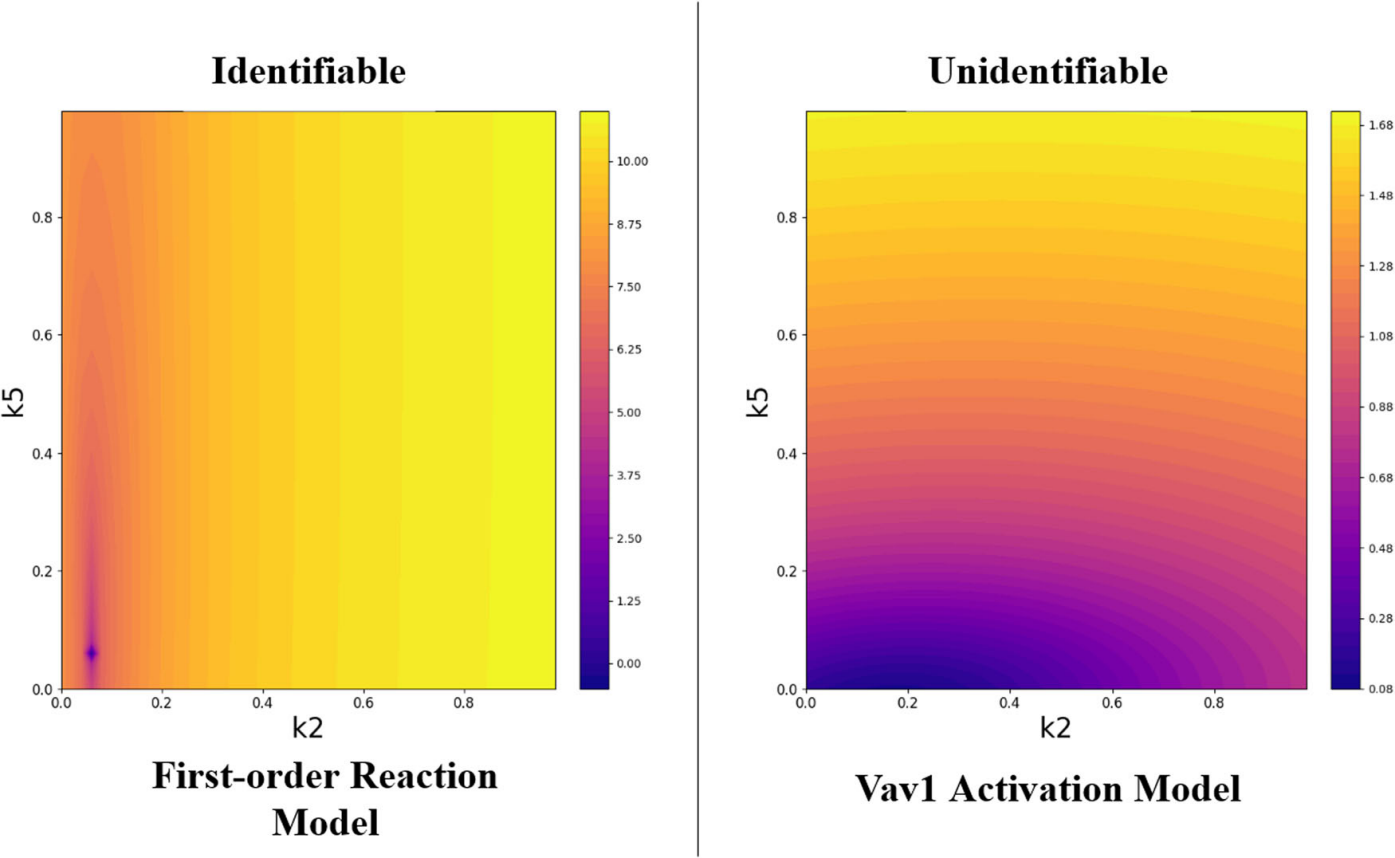
Contour plots for identifiability. Contour plots of the landscape of the cost function are shown as a function of k_2_ on the *x* axis and k_5_ on the *y* axis with all other model parameters held constant at the ground truth. The left contour plot is a pairwise contour plot of the fully “identifiable” first-order reaction system where a steeply convex cost landscape surrounds the ground truth parameters at the bottom left. The right pairwise contour plot is of the nonlinear Vav1 activation model where a nonconvex cost landscape results in the parameter estimates shown in Fig. 7. Note the big difference in the scale of the color values for the two plots. The color bar is of the log-GMM cost that is derived by BioNetGMMFit with 6 means, 6 variances, and 15 covariances in its calculation.

We acknowledge the previous work of Sethna et al.^26^, which used information theory to characterize parameter identifiability issues and model sloppiness; and the work of Sorger et al.^27^, which used a Bayesian approach to compare competing models in the presence of partial identifiability. Although BioNetGMMFit is not designed to directly address those issues, our software does give users the ability to inspect a given model for identifiability concerns by constructing pairwise contour plots of the high-dimensional cost (aka objective) function. For each contour plot the x and y axes correspond to a pair of distinct rate constants. For example, pairwise contour plots can be generated for the nonlinear Vav1 activation model, where local minima were virtually indistinguishable from the ground truth, resulting in parameter estimates that were different from the ground truth (see Fig. 7). By creating pairwise contour plots for k_2_ (or *θ*_2_) and k_5_, where PSO was unable to discern the ground truth values (see Fig. 8), a striped pattern on the log-cost contour plot reveals a region of unidentifiability. In contrast, the well-estimated first-order reaction model has a convex log-cost shape (see Fig. 8).

### Comparison with other software tools

**Fig. 9:**
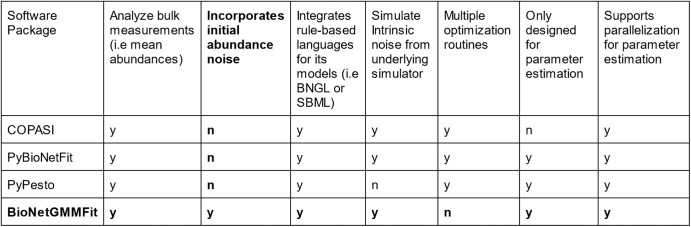
Comparison of BioNetGMMFit features. Bulk measurements refer to measurements in which one obtains a single observed value for each species of reactants, at each time point. The observed value may be the mean or total abundance of the species at that time point. In contrast, single-cell measurements yield copy numbers or concentrations of each species for thousands of cells at each time point. This leads to what we refer to as initial abundance noise. The letter “y” indicates that the feature is available and “n” implies that the software is not designed to do it.

By combining the best aspects of CyGMM and BioNetGen, BioNetGMMFit provides several noteworthy features for the analysis of single-cell time-stamped cytometry data that complements the currently available parameter estimation software suite (i.e PyBioNetFit^18^, Statistical Model Checking (SMC)^28^, COPASI^29^). In particular, BioNetGMMFit offers the ability to efficiently analyze single-cell trajectories for a large number ( > 1000) of cells, predict higher-order moments at future times, and simulate parameter estimation tasks with ground truth parameters. We detail the full capabilities and limitations of BioNetGMMFit below as well as how it compares to other pre-existing parameter estimation software packages. A brief summary of the software-related differences are shown in Fig. 9.

#### Dealing efficiently with cell-specific initial conditions

BioNetGMMFit accurately and efficiently estimate BioNetGen model parameters from cytometry snapshot data observed across many single-cell trajectories. It employs the same sophisticated minimization procedure as CyGMM^7^ to estimate parameters from the analysis of higher-order moments. To the best of our knowledge, existing software are not designed to account for large variances in initial conditions. Thus, they have difficulty performing parameter estimation efficiently where (1) initial abundance noise is known to play a role and (2) the data consists of large numbers of different single cells (where each cell has its own set of trajectories).

For example, when the estimation depends on the analysis of data from a large number of single cells (as opposed to bulk measurements averaged over cells), existing software is either not designed to handle such data^18,28^, or is less user-friendly, or is computationally inefficient^14^. In many cases, when attempting to perform parameter estimation across a combination of moment statistics, inefficiencies related to file input and output occur. For instance, in using BioNetGen’s simulator, each cell has its own BioNetGen file containing a bulk measurement such as average protein abundances. Therefore, to perform parameter estimation across a combination of different measurements (i.e., means, variances, and covariances) with BioNetGen only^14^, the user must repeatedly read from multiple data files in its parameter estimation. In PyBioNetFit^18^, only a single set of cell conditions per time point are used for parameter estimation. Our method allows users to input easily several time-stamped snapshot files containing abundance data from thousands of cells and store them directly in memory for quick simulations. See Supplementary Fig. 10 below for each dataset’s cell count used for parameter estimation. While the comparison is not exact as the models and objective function (i.e BioNetGMMFit’s incorporation of higher-order moments) analyzed were different, we show that we can attain run-time performance comparable to that of PyBioNetFit^18^. In the analysis of the Vav1 activation model in Fig. 6 with 5000 single cells, the run time was ~1.9 h on a 16-core processor for 30 repeated parameter estimates, which is in the range of run time using the SBML simulator in PyBioNetFit for a similar number of cores^18^. To provide some context on run time, we compare with a method such as SMC^28^: while again the comparisons are not exact as the methods and models are different, our run times are comparable to their 4.2 h for parameter estimation. Due to C++ parallelization with OpenMP, our software is capable of linearly scaling with increasing cell count, thereby reducing run times for large numbers of cells. Supplementary Fig. 12 provides run-time details concerning the number of cells and model parameters.

#### Estimating with higher-order moments

Due to their design intended for bulk measurements, existing software such as PyBioNetFit^18^, COPASI^29^, and PyPesto (from the PETab suite)^30^ for rule-based parameter estimation are not specifically designed to fit higher moments. While possible to do a plethora of file input and output to generate multiple parameter distributions per set of initial conditions and generate moment statistics using PyBioNetFit or BioNetGen, for a large number of cells, parameter estimation becomes very user-unfriendly and computationally taxing. In contrast, BioNetGMMFit directly supports three levels of analysis corresponding to using an increasing number of moments. First, one can choose to estimate parameters using only the means (i.e., first moments). Second, one can use both means and variances or third, use means, variances, and covariances. Results for moments with different levels of fitting are shown with its different columns in Fig. 3.

Other more mature softwares such as COPASI^29^ offer a wide variety of other features such as an executable graphical user interface, the ability to export ODEs from these rule-based languages, as well as the support of a wide range of optimization routines. For optimization routines involving smaller and simpler (i.e., linear instead of nonlinear) models where large initial abundance noise is not of concern, it is preferable to use COPASI^29^, PyBioNetFit^18^, and PyPesto^30^ due to the availability of less computationally expensive gradient-based optimization routines. However, in such simple parameter estimation tasks, BioNetGMMFit performs functionally the same due to its flexibility of cost functions where the user can simply choose to use only the observed first moments.

## Discussion

We present a new software: BioNetGMMFit, that greatly enhances a researcher’s ability to explore a wide range of mechanistic models while using higher-order moments to leverage the information in single-cell data. In particular, BioNetGMMFit provides accurate estimation of model parameters, and reports valid confidence intervals for each estimated parameter as well. Furthermore, BioNetGMMFit can easily handle data observed across multiple time points, and it can predict protein abundances at future times. A key feature of BioNetGMMFit, which benefits from its integration with libRoadRunner’s simulators, is its ability to easily simulate protein abundances at scale (i.e., across multiple time points, across many single cells, and under a wide variety of different biochemical signaling models). Moreover, because such simulations can now easily be carried out at scale, the ability to tune hyperparameters has become a much more accessible task.

Hyperparameter tuning involves selecting the optimal set of hyperparameters of the optimization routine to achieve the best parameter estimates that minimize the cost function and enable optimal prediction from one’s model. For example, in the case of PSO, there are five hyperparameters to tune, including the number of particles, the number of steps, and three coefficients that affect how the particle swarm moves in the space of model parameters. To address this challenge, we use a simulated reference point of the “optimal” or ground truth parameters for a BioNetGen model, which can provide insights into the performance of an optimization routine’s hyperparameter configuration. A practical way of determining a suitable set of hyperparameters when analyzing experimental data is as follows. If one wishes to fit a model to the data, one can simulate a ground truth version of the model separately and adjust the hyperparameters so that BioNetGMMFit estimates the ground truth values to desired accuracy. Then, one can use these newfound hyperparameters to fit the model to the experimental data.

### Limitations of BioNetGMMFit

BioNetGMMFit is currently limited to parameter estimation for well-mixed mechanistic models describing deterministic and stochastic kinetics. Unlike PyBioNetFit^18^, other forms of simulation such as NFsim^31^ and spatial modeling^32,33^ are not supported. Furthermore, BioNetGMMFit does not support any optimization algorithms other than PSO. In addition, the lack of Python support may make it difficult to integrate it with pre-existing workflows that rely heavily on Python. While there exists a C++ static binary that has been tested on Ubuntu Linux, compilation with libRoadRunner C++ libraries can be a nontrivial process on other Linux distributions. Thus, BioNetGMMFit is most readily accessible through Docker, which although accessible across all major operating systems, still requires the use of third-party virtualization software. These software weaknesses will need to be addressed in future iterations of the software.

## Methods

### Software inputs

**Fig. 10:**
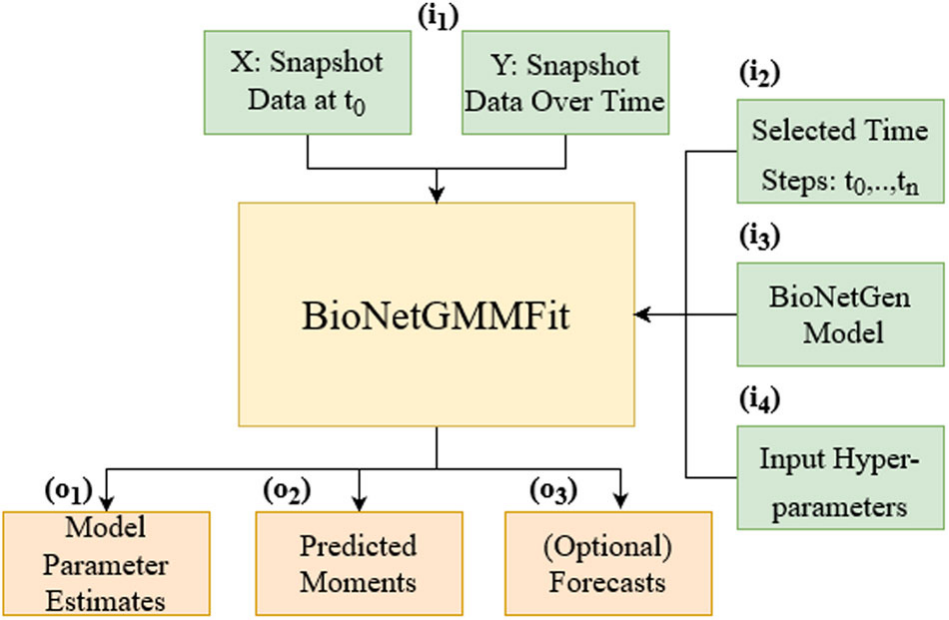
BioNetGMMFit inputs and outputs. The inputs and outputs to the program BioNetGMMFit (yellow box) are displayed. The green boxes, labeled with the letter “i'', are the inputs to the program. The two boxes included in i_1_ represent the input directories: X contains the initial conditions for the model and Y, all the observed snapshot data at different time points that must be passed to BioNetGMMFit. The box i_2_ is the time steps file that contains all the specified time points at which the concentrations are evaluated, by evolving the model given the initial conditions. i_3_ represents the model defined in BioNetGen containing the parameters to be estimated. The BioNetGen model (defined in BNGL) is used to evolve the initial conditions in X to fit the observed moments computed from the data in Y. i_4_ is the configuration file that contains the hyperparameters of the PSO (i.e the number of particles, steps, and PSO weights) that need to be defined by the user for the parameter estimation task. The orange boxes indicate outputs and are labeled by “o''. BioNetGMMFit produces two explicit outputs, o_1_, the parameter estimates of the BioNetGen model and o_2_, the corresponding time-evolved moments. The moments in o_2_ are generated using the parameter estimates that minimize the GMM cost between the observed moments and predicted moments. Lastly, the user can choose to forecast future trajectories of moments in o_3_.

**Fig. 11:**
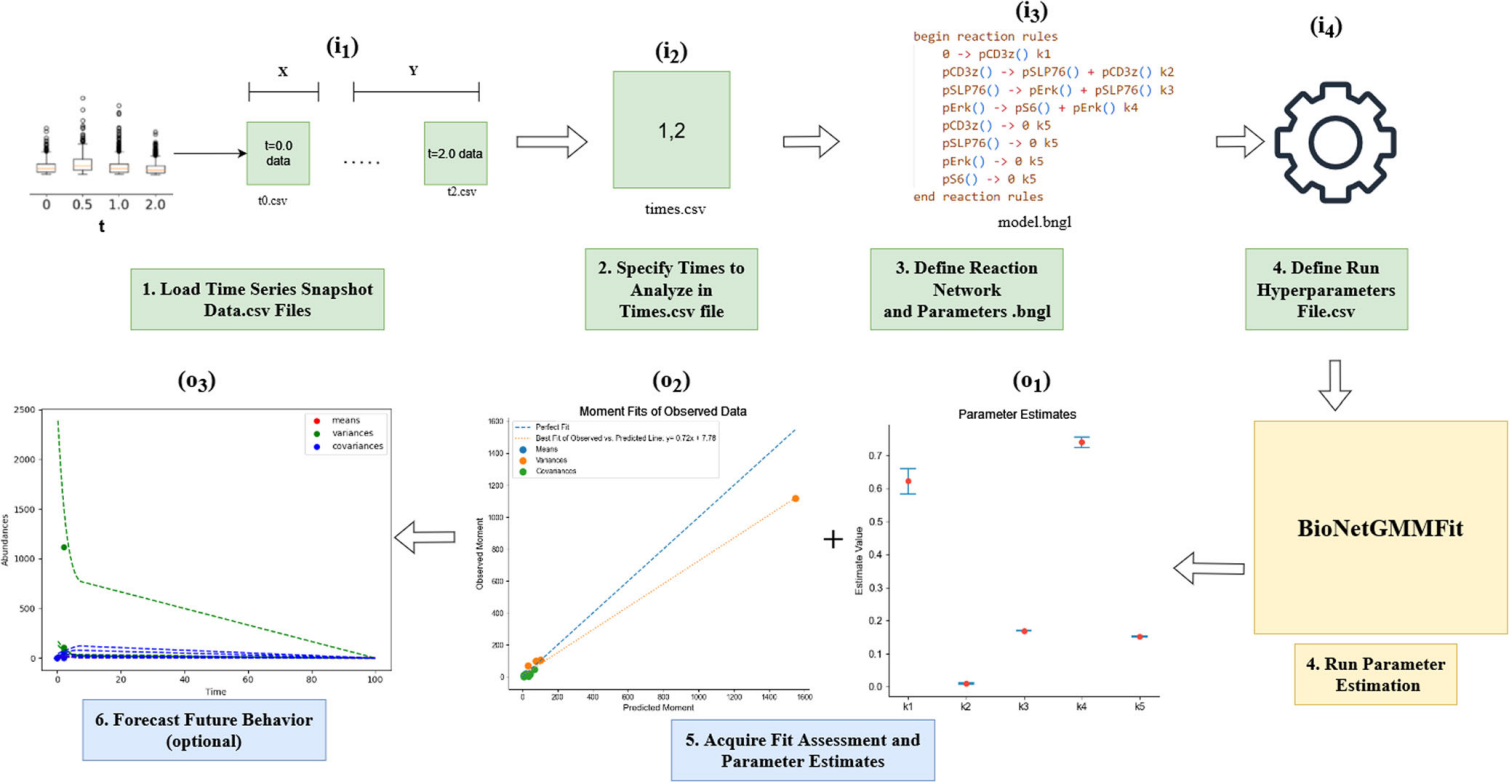
BioNetGMMFit workflow. All “i’’’s and “o’’’s correspond to the same inputs and outputs defined in Fig. 10. In this figure, we depict the steps needed to run BioNetGMMFit relating back to the inputs and outputs of Fig. 10. To begin, the snapshot data of interest must be loaded by first organizing all .csv files into their corresponding initial conditions (X) and observed conditions (Y) directories, and then these (X) and (Y) directories (i_1_) must be specified to BioNetGMMFit. The black points in the data boxplot figure represents the outlier of the dataset (specifically CD8+ T-cell dataset). Next, the user must define the time points that correspond to each of the snapshot files by creating and specifying a times.csv file (i_2_). Once all of the data are defined, the user must define and specify the BioNetGen model of interest (i_3_) to BioNetGMMFit. The last input required is the hyperparameters configuration file where the user must define all of the necessary parameters for a parameter estimation run, specifically related to the PSO algorithm that will be used to estimate the BioNetGen model parameters. Once all necessary inputs (i_1_, i_2_, i_3_, and i_4_) are defined, BioNetGMMFit will run its parameter estimation routine with GMM and PSO, returning parameter estimates (o_1_) (and confidence intervals if there are multiple replicate runs of PSO) as well as a *observed* = *predicted* line where observed moment values are on the *y* axis and predicted moments (o_2_) generated from the estimated model parameter on the x axis. Please note that the observed moments are calculated from the abundance data supplied by i_1_, and similarly the predicted moments are computed from the evolved abundances generated by simulating each initial sample with the BioNetGen model. Finally, while optional, users of BioNetGMMFit can predict moments of future time points (o_3_) of the BioNetGen model. Please refer to the Supplementary Material for a more in-depth tutorial on how to use the command line version of BioNetGMMFit illustrated with the CD8+ T-cell dataset.

To illustrate in detail how one might use BioNetGMMFit, a parameter estimation task with an experimental CD8+ T-cell dataset was performed. As shown in Fig. 10, BioNetGMMFit reads input data from several sources, including (i_1_) a directory containing a CSV file with initial protein abundances (X) and a directory containing CSV files with observed protein abundances at different time snapshots (Y); (i_2_) a time steps CSV file that contains the times at which the data of interest are observed (note that the data of interest may be a subset of the full data); (i_3_) a .bngl (BioNetGen) file that describes the mechanistic model capable of executing deterministic or stochastic kinetics; and (i_4_) a BioNetGMMFit hyperparameter configuration file that enables various features, such as generating time-series snapshot data from the BioNetGen model or changing the number of particles and steps of the PSO routine. A general overview of the BioNetGMMFit workflow is illustrated in Fig. 11.

When working with directories of data files, only one CSV data file is read from directory X to establish the initial set of protein abundances. Meanwhile, directory Y may contain multiple CSV files for each time step being analyzed. These data files are arranged such that each row contains the observations (i.e., protein abundances) from an individual (or single) cell. To ensure that protein abundances or concentrations are nonnegative, any single-cell data containing negative protein values at a given time is removed before performing parameter estimation in BioNetGMMFit. Such a preprocessing step reduces the necessary steps the user must take to properly use BioNetGMMFit, as negative values are commonly generated in CyTOF datasets during a processing step that spreads out zero readings in CyTOF measurements^34^.

### Working example

**Fig. 12:**
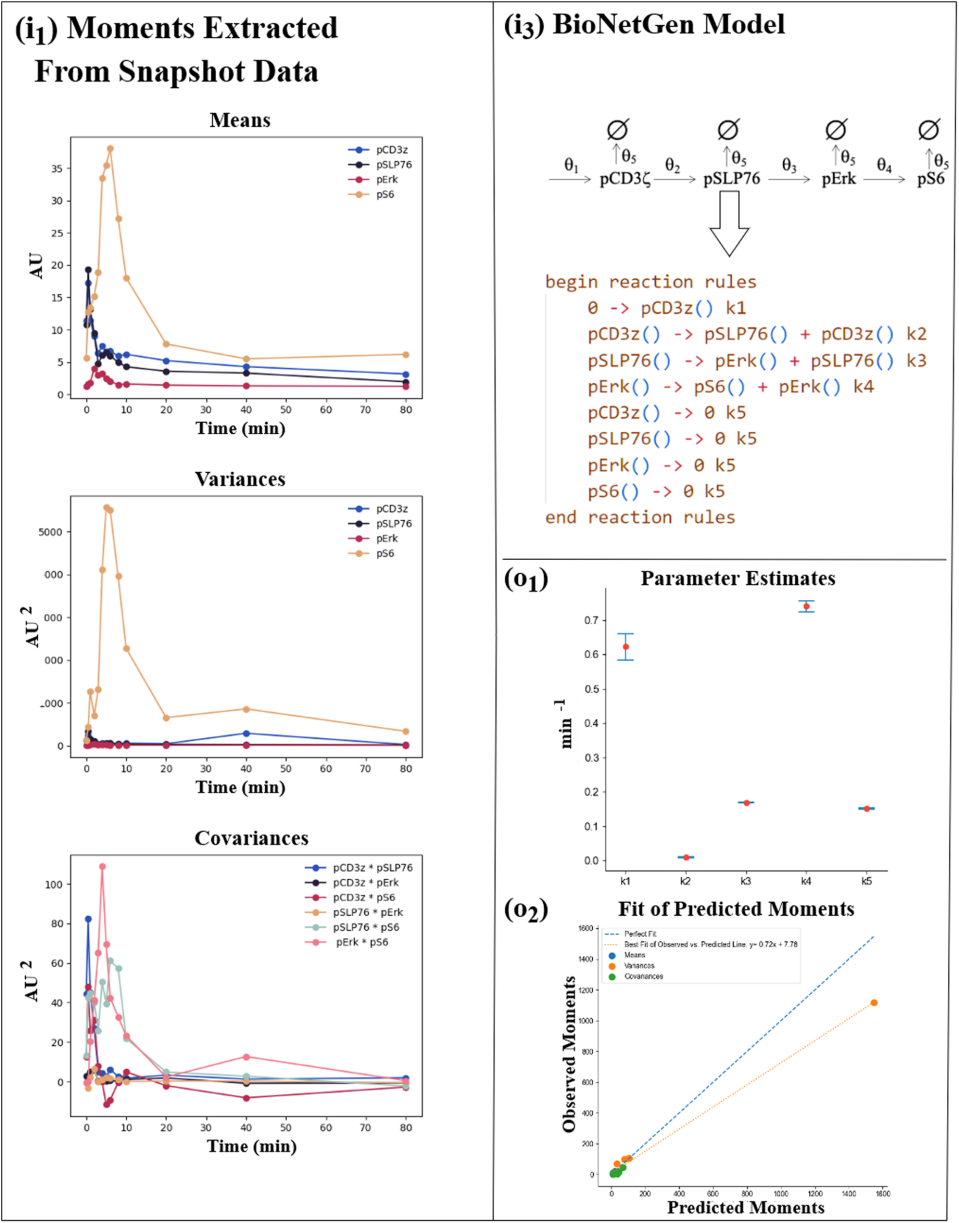
CD8+ T-cell example. The input and output results for fitting the CD8+ T-cell CyTOF dataset are shown. The panel labels (i_1_), (i_3_), (o_1_), and (o_2_) correspond to those in Fig. 10. Panel (i_1_) in Fig. 12 shows the sample means, variances, and covariances of CD8+ T-cell proteins calculated by BioNetGMMFit for the observed time points of snapshot data (supplied in the files i_1_ and i_2_). These are used for parameter estimation as part of the GMM procedure. (More information on GMM can be found in the Supplementary Material.) Panel (i_3_) displays the reaction network and its corresponding reaction rules in the .bngl file. Each reaction rule represents a reaction within the model and has a parameter (rate constant) associated with it (e.g., k1). Using the observed moments (i_1_ panels) BioNetGMMFit uses PSO and GMM to estimate parameters of the model. (o_1_) and (o_2_) illustrate the results for a case where BioNetGMMFit performs 30 PSO estimates and produces a set of confidence intervals as well as a plot of the least GMM cost fit between the observed moments and predicted moments. The fit is performed with 4 protein means, 4 protein variances, and 6 protein covariances. Please note that the user is responsible for the consistency of units units in the input data since BioNetGMMFit uses the input value without ascribing about measurement units. For the CD8+ T-cell data used above, the input data were of the magnitude of fluorescence.

As an example, we will use a signaling kinetic model involving four proteins to analyze a CD8+ T-cell CyTOF dataset. One biological question of interest that can be addressed with such data is to quantify the rate of the signal propagation between a pair of signaling proteins and study how the rates depend on the developmental state of the immune cell (e.g., naive vs memory CD8+ T cell). We applied BioNetGMMFit to describe signaling kinetics in CD8+ T cells stimulated by CD3 and CD28 antibodies where binding of the antibodies induce phosphorylation of the transmembrane CD3*ζ* chains which further lead to phosphorylation of the adaptor protein SLP76, MAPK kinase Erk and the ribosomal protein S6 following pCD3*ζ* → pSLP76 → pErk → pS6^35^. We model the phosphorylation reactions shown above by first-order reactions. There are many intermediate biochemical reactions involved within the phosphorylation steps shown above and we assume the rates of the first-order reactions effectively capture the effect of those intermediate reactions. The phosphorylated protein species also go through degradation/ubiquitylation processes which are also approximated by first-order decay reactions. In addition, pCD3*ζ* is assumed to be produced at a constant rate due to the signaling process. The model contains five rate constants, *θ*_1_, ⋅ ⋅ ⋅ , *θ*_5_, which we estimate using BioNetGMMFit. BioNetGMMFit also plots confidence intervals as well as the observed and estimated moments as shown in Fig. 12. The underlying datasets used in this parameter estimation task of the CD8+ T cells are from (https://dpeerlab.github.io/dpeerlab-website/dremi-data.html) ^35^. From this dataset, one can compute time trajectories of the protein moments, means, variances, and covariances (see Fig. 12). To make the presentation simple, we will only analyze trajectories at two time points: 1 min and 2 min. In this case, 719 cells were measured at time point 1 in and 653 cells were measured at time point 2 min. Again, as shown in Fig. 12, mechanistic model reaction rules are defined in the .bngl file where they are then converted into an SBML file using BioNetGen’s writeSBML() function as BioNetGen does not support its own C++ API. Using libRoadRunner’s C++ API^15^, BioNetGMMFit converts this SBML file into a roadrunner object that is capable of simulating the defined model to user-specified time intervals. While there are many other reaction network simulators such as PySB^36^, COPASI^29^, and AMICI^37^, libRoadRunner was primarily chosen for its speed and ease of customizability due to its function as a C++ API rather than a standalone software package.

Once the model is defined, the hyperparameters configuration file supplies the PSO hyperparameters (i.e., the number of particles, steps, and PSO coefficients) used for parameter estimation. For more information on the configuration file, a table of parameters is provided in the documentation on the GitHub page and in the Supplementary Material. To estimate parameters within the BioNetGen model, PSO minimizes the GMM-weighted square Euclidean distances of lower and higher-order moments between the observed and model-simulated data^7^. Upon PSO convergence, estimates are provided, and confidence intervals are computed.

The plots of parameter estimates and moment fits provide additional insights into the fit of the mechanistic model. In Fig. 12, for example, the confidence intervals around the parameter estimates reveal the ranges of possible values for each model parameter and their relative magnitudes. The estimation shows that the rates of production of pCD3*ζ* and of pS6 induced by pErk are the two dominant signaling reactions that occur at the early times (1–2 min) when CD8+ T cells are stimulated. This result is consistent with related conclusions in ref. ^35^ obtained using information theory. Nonetheless, models are imperfect, and their fits may be poor. To assess the model fit in such cases, we can refer to the “Fit of Predicted Moments” plot in Fig. 12. This plot shows that the estimated model fails to accurately fit one of the protein’s variances, as indicated by a variance dot biased below the perfect fit line, indicating that further modifications in the model are needed to better fit the data. Such moment plots are essential for evaluating the parameter estimation and model fits that may be difficult to observe with only mean or bulk abundance differences.

BioNetGMMFit is currently available as a docker image for ease of portability across different operating systems, a compilable executable for those requiring high-performance computing capabilities (https://zenodo.org/record/7733865), and as a web demo (https://bngmm.nchigm.org/). Further details on how to use and compile BioNetGMMFit are available on the GitHub page (https://github.com/jhnwu3/BioNetGMMFit), and a comprehensive tutorial on using BioNetGMMFit can be found in the Supplementary Material.

### Reporting summary

Further information on research design is available in the Nature Research Reporting Summary linked to this article.

### Supplementary information

Supplementary Material - BioNetGMMFit

Reporting Summary

## Data Availability

The dataset(s) supporting the conclusions of this article is(are) available in the BioNetGMMFit/example repository [https://github.com/jhnwu3/BioNetGMMFit/tree/main/example]. Project name: BioNetGMMFit; Project home page: https://github.com/jhnwu3/BioNetGMMFit; Archived version: N/A; Operating system(s): Platform independent through Docker; Programming language: C/C++; Other requirements: Docker, if Compiling see GitHub; License: MIT License; Any restrictions to use by non-academics: N/A; Other Locations of Access: https://hub.docker.com/repository/docker/jhnwu3/bngmm/general, https://zenodo.org/record/7733865, https://bngmm.nchigm.org/. Experimental CD8+ T-cell data can be found at https://dpeerlab.github.io/dpeerlab-website/dremi-data.html.
