# Supplementary material for: BioNetGMMFit: estimating parameters of a BioNetGen model from time-stamped snapshots of single cells
Source: NPJ Syst Biol Appl. 2023 Sep 22;9:46. doi: 10.1038/s41540-023-00299-0 (PMC10516955; doi:10.1038/s41540-023-00299-0)
Supplement: Supplementary file 1 — Supplementary Material - BioNetGMMFit [file 41540_2023_299_MOESM1_ESM.pdf]

Supplementary Notes

GMM Primer

Here, we give a short and simple exposition of MOM (method of moments) to motivate and describe its extension: GMM (generalized method of moments). Consider data $D_1, \ldots, D_n$, where each D_i is independent and identically distributed $N(\mu, \sigma^2)$. MOM equates sample moments such as $\overline{D}$ and $\overline{D^2}$ to their corresponding expected values (μ and $\sigma^2 + \mu^2$). Under a mild set of conditions, these equations can be solved yielding consistent estimates ($\hat{\mu}$ and $\hat{\sigma}$) of the unknown parameters (μ and σ). Now, to illustrate how important MOM can be, let's consider another simple example. Suppose we have data Z_i where each Z_i is distributed $Beta(\alpha, \beta)$. We can define the jth sample moment (denote m_j) as $1/n \sum {Z_i}^j$. Now, using the first and second sample moments, MOM yields the following equations: $\overline{Z} = \alpha/(\alpha+\beta)$, and $\overline{Z^2} = \alpha/(\alpha+\beta)^2[\beta/(\alpha+\beta+1)+\alpha]$. With two equations, and two unknowns, estimates $\hat{\alpha}$ and $\hat{\beta}$ are the closed form solutions for α and β given in terms of the sample moments m_1 and m_2. Note that for this simple example one must resort to numerical methods to find the maximum likelihood estimates of α and β. Furthermore, as there are an infinite number of moments (and sample moments), the decision to use m_1 and m_2 is somewhat arbitrary. In practice since expectation values of lower order moments have lower errors for finite size datasets, lower order moments (e.g., means, variances, covariances) are usually considered for parameter estimations.

GMM (generalized method of moments) is a generalization of MOM that yields consistent estimation of θ (i.e. a vector of unknown parameters) when the number of moment equations (*aka* moment conditions) is larger than the dimension of θ (i.e. the number of unknown parameters). In this manuscript we have snapshot data observed at multiple time points, but for simplicity, let's consider snapshot data X observed at time 0 and snapshot data Y observed at time t. The sample moments in Y are then compared to their corresponding expected values, which are approximated by first

evolving X to time t for a given θ [denoted h(X;θ, t)] and then computing the sample moments of h(X;θ,t)]. Because the system of equations is often overdetermined, there typically isn't a solution (i.e. no value of θ makes the difference between sample moments exactly zero). Therefore, the GMM approach advocates finding the value of θ that *minimizes* the distance between the sample moments of Y and the sample moments of h(X;θ,t)]. Let's denote this k-dimensional vector of differences by $\Delta m(\theta)$, so that the distance between sample moments is simply $[\Delta m(\theta))W(\Delta m(\theta)]^T$. For example, when W is the $[k$ x $k]$ identity matrix, the GMM cost is the usual geodesic distance in $\mathcal{R}^k$ which also corresponds to a *least squares approach* and has been shown to perform poorly compared to the GMM cost that uses the optimal W (7). In practice, one often begins with W_1 equal to the identity matrix, and then minimizes $[\Delta m(\theta)]W_1[\Delta m(\theta)^T]$ to obtain the first estimate of the unknown parameters, denoted $\hat{\theta}_1$. Then, one typically estimates $W_2 = \left(E\left[\Delta m^T(\hat{\theta_1})\,\Delta m(\hat{\theta_1})\right]\right)^{-1}$ because, in accordance with (Hansen et al. (4)), the next iterate $\hat{\theta_2}$ is obtained by minimizing $[\Delta m(\theta)]W_2[\Delta m(\theta)^T]$. This process can be repeated for any number of steps; but for the standard two-step estimator, one simply stops at $\hat{\theta_2}$. Note that W_2 involves matrix inversion, which is often done numerically. Finally, in order to find the value of θ that minimizes $[\Delta m(\theta)]W[\Delta m(\theta)^T]$, one should perform an efficient search over the parameter space. In particular, for the problems discussed in this manuscript (and for most problems in general), a brute force grid search is computationally infeasible. Fortunately, there are many such optimization routines to choose from, and we find that particle swarm optimization (PSO), which is amenable to parallel computation, works well even when the dimension of θ is large, and the distance function $[\Delta m(\theta)]W[\Delta m(\theta)^T]$ is multi-modal.

For a practical tutorial on GMM, please see our Python tutorial (https://github.com/jhnwu3/BioNetGMMFit/blob/main/example/gmm_tutorial.ipynb).

BioNetGMMFit Tutorial for CD8+ T cell Dataset

The data we are looking at is a set of snapshot data files across different time points. Such snapshots across time are shown below as box plots in Figure 1.

First, let us consider the command line call in Figure 2.

Inside this call, there are multiple required command line arguments that are explained in Figure 3 below.

Taking a look at the .bngl file in Figure 4, there are several important caveats. The observables list also define the order in which data must be organized in its respective data csv files where the top observable matches the first column of the csv data files. Likewise, the order of the list of the parameters matches the vector that will be estimated. Please also note that the .bngl file has to write to SBML for BioNetGMMFit to work.

Zooming in on the data directories and time steps file, please note that the X directory contains specifically a csv file containing the initial conditions of the snapshot of CD8+ T cells at time t=1 minute. Again, the Y directory contains other csv files of the observed snapshot data at future time points. In the case of usage, the user should make sure that each Y directory file is named the same and has the time point number at the end of the file name as BioNetGMMFit reads in each file alphanumerically such that file with the smallest time point in its name corresponds to the first future time observed in the time steps csv file. A diagram of X and Y directories along with a set of time steps from the time steps file is shown in Figure 5. In this scenario, since there are only two time points, the initial conditions is considered to be at time t=1.0 minutes and the final observed conditions is at time t=2.0 minutes, and this corresponds to only one file being in the initial conditions directory and the other file being in the other observed conditions directory.

A BioNetGMMFit parameter estimation run is then characterized by its configuration file, which defines several key PSO hyperparameters such as the number

of particles and steps. An example is given in Figure 6. All parameters in the configuration csv file are explained in Figure 7.

Once the parameter estimation run begins, the command line program will output information such as the weight matrices computed from the snapshot data and observed moments. The number of weight matrices correspond to the number of snapshot csv files in the Y directory. Once the parameter estimation finishes, estimates and their corresponding costs are outputted as shown in Figure 8. Additionally, plots of moment fits and their 95 % confidence intervals are also generated as displayed in Figure 9. Should the user want to, forecasting can also be performed with the mean parameter estimate of their model using "-f futureTimesFile.csv" as part of the command line call.

Again, for more information and details on the example along with a UI parameter estimation counterpart, please see the GitHub repo (https://github.com/jhnwu3/BioNetGMMFit).

Computational Costs

Run times may vary drastically depending on the time points, models, and numbers of single cells being analyzed. To provide some idea of computational costs, we profiled the CD8+ T cell signaling model and the nonlinear signaling model shown in Figure ??. The number of model parameters, PSO hyperparameters, and dataset characteristics are shown in Figures 11 and 10 while their respective run times are shown in Figure 12. Please note that each run is considered a PSO estimate. To generate confidence intervals, more than one run is required while generally 30 or more runs are preferred. All run times are rounded to the nearest second and all runs were performed using a 16-Core AMD EPYC 7302 processor with 32 threads and a maximum clock speed of 3.3 GHz. Only 30 threads were used for testing.

Although their asymptotic relationship may differ depending on the BioNetGen model, increasing the particle swarm's number of particles and step count generally has a linear effect on run time where a 10× in particles tends to lead to a 10× in run time. In terms of data inputs, increasing the number of cells has a linear relationship with run time. More complex models have a dramatic effect on simulation time where increasing the number of parameters and species in a system increases run time in a nonlinear fashion. Similarly, as ode-solvers must approximate solutions, increasing the time evolutions tend to have a nonlinear relationship with run time. Fortunately, as BioNetGMMFit is parallelized through OpenMP, users can reduce run time by increasing the number of cores accessed by BioNetGMMFit.

4.3 Supplementary Figures

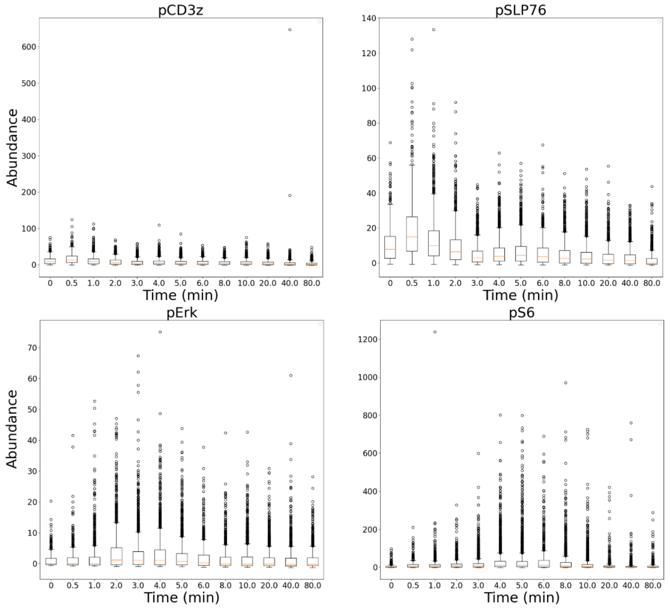

Supplementary Figure 1 CD8+ T cell Snapshot Data: Black circles indicate points outside of the quartiles i.e outliers in the data. Please note that the x-axis is not relative to scale of time points.

```
jhnwu3@JW:/mnt/c/Project_Folder/Cpp/CyGMM_DockerBuild/BNGMM$ ./BNGMM -m example/4_prot_
CD3_CD8_CD28/4proV2.bngl -c example/4_prot_CD3_CD8_CD28/Config4pro.csv -x example/4_pro
t_CD3_CD8_CD28/1min_2min/X/ -y example/4_prot_CD3_CD8_CD28/1min_2min/Y/ -t example/4_pr
ot_CD3_CD8_CD28/time_steps.csv -o test/
```

Supplementary Figure 2 BNGMM Command Line Call

Command Line Argument	Explanation
`-m example/4_prot_CD3_CD8_CD28/4proV2.bngl`	Defines the path to model .bngl
`-c example/4_prot_CD3_CD8_CD28/Config4pro.csv`	Defines the path to the PSO run configuration file
`-x example/4_prot_CD3_CD8_CD28/1min_2min/X/`	Defines the path to the “X” initial conditions directory
`-y example/4_prot_CD3_CD8_CD28/1min_2min/Y/`	Defines the path to the “Y” observed evolved snapshot conditions directory
`-t example/4_prot_CD3_CD8_CD28/time_steps.csv`	Defines the path to the time steps csv file
`-o test/`	Defines the output directory that contains all of the file outputs that is generated by BioNetGMMFit or BNGMM
`-f future_time_steps.csv`	Defines the path to the future time steps file that will be used to generate moments at specified time points. (optional)
`-contour k1 k2`	Will enable the generation of pairwise colour/contour plots of k1 and k2 (optional)

Supplementary Figure 3 BNGMM Command Line Arguments

```
begin model

begin parameters
    k1 0.1
    k2 0.1
    k3 0.95
    k4 0.17
    k5 0.05
end parameters
begin species
   pCD3z() 192.7959
   pSLP76() 1463.265
   pErk() 5.251
   pS6() 435.2968
end species

begin observables
    Molecules pCD3z pCD3z()
    Molecules pSLP7 pSLP76()
    Molecules pErk pErk()
    Molecules pS6 pS6()
end observables

begin reaction rules
    0 -> pCD3z() k1
    pCD3z() -> pSLP76() + pCD3z() k2
    pSLP76() -> pErk() + pSLP76() k3
    pErk() -> pS6() + pErk() k4
    pCD3z() -> 0 k5
    pSLP76() -> 0 k5
    pErk() -> 0 k5
    pS6() -> 0 k5
end reaction rules

end model

generate_network()
writeSBML()
```

Supplementary Figure 4 CD8+ T cell BioNetGen Model File

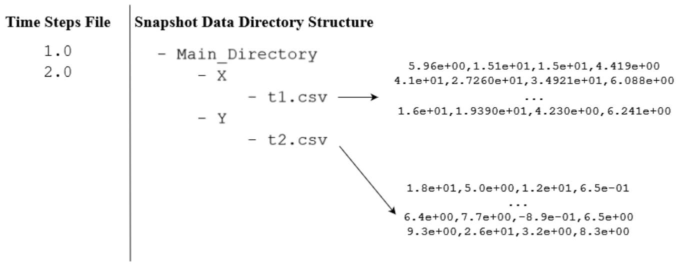

Supplementary Figure 5 BioNetGMMFit X and Y directory structures

Example Config.csv used

```
Number of Particles PSO,150
Number of Steps PSO,25
Exclude Mixed Moments?,1
Exclude Mixed and Second Moments?,0
Number of Runs,25
Simulate Y_t?,-1
Use Matrix Inverse?,-1
Number of Rates,5
Hypercube Dimension,1
Report Moments?,1
Bootstrap?,0
Use Deterministic?,1
Number of BNGL Steps,1
Seed,-1
ParallelNumberOfThreads,8
Initial Particle Best Weight, 3.0
Initial Global Best Weight, 1.0
Particle Inertial Weight, 6.0
```

Supplementary Figure 6 BioNetGMMFit Configuration File Example

Parameter	Value	Explanation
Number of Particles PSO	1000	Sets number of particles in PSO
Number of Steps PSO	10	Sets number of steps for PSO
Exclude Mixed Moments?	0	1 to use only means and variances, 0 otherwise
Exclude Mixed and Second Moments?	0	1 to use only means, 0 otherwise
Number of Runs	1	Sets total number of PSO runs for estimation
Simulate Y_t?	1	1 to simulate Yt with a true rate vector, 0 to provide own Yt matrix
Use Matrix Inverse?	0	1 to use C++'s Matrix Inverse, 0 otherwise
Number of Rates	5	Sets number of parameters to be estimated
Hypercube Dimension	1.0	Real Value Bounds of Hypercube to be searched in PSO.
Report Moments?	1	1 to report predicted moments in out.txt
Bootstrap?	1	1 to estimate 95% CI's, 0 otherwise
Use Deterministic?	1	1 to use CVode integrators, 0 to use roadrunner gillespie simulation
Number of BNGL Steps	15	Tuning Parameter for number of steps of integration
Seed	-1	Used to seed the PSO, Off when seed < 0, On when seed > 0
Parallel Number of Threads	8	Number of threads to parallelize on.
Initial Particle Best Weight	3.0	How much historical weight (i.e last known particle position) to affect PSO step.
Global Best Weight	1.0	How much weight best particle affects next PSO Step.
Particle Inertial Weight	6.0	PSO Particle Inertia Component (to avoid local minima)

Supplementary Figure 7 BioNetGMMFit Configuration File Parameters Explained

```
--------------- All Run Estimates: -------------------
k1 k2 k3 k4 k5 cost
  0.507163 0.00398043   0.201055   0.730855   0.142193  0.0204464
  0.648754 0.00239177   0.205644   0.759204    0.14769  0.0200926
  0.703837   0.012705   0.207977   0.809088   0.155427  0.0206755
   0.44794 0.00312797   0.202042   0.722827   0.141108  0.0206706
  0.313449 0.00338228   0.196831   0.694171   0.134851  0.0219995
  0.474989 0.00522015   0.201166   0.718512    0.14247  0.0207732
     0.805  0.0173536   0.213123   0.825059   0.161618  0.0211036
   0.58846  0.0051885   0.204561   0.746665   0.147583  0.0202742
   0.61945 0.00526015   0.206712   0.748316     0.1481  0.0202485
  0.407597  0.0024846   0.198855   0.724296   0.137528  0.0208935
  0.625188 0.00511577   0.207393    0.72513   0.148294  0.0204046
  0.721672  0.0100798   0.210919   0.803797    0.15637  0.0206155
  0.700379  0.0102639   0.207832   0.786764    0.15406  0.0204902
  0.638543 0.00997491    0.20782   0.796317   0.152459  0.0205387
  0.452629 0.00380433   0.202921   0.718286   0.140688  0.0207769
  0.577947  0.0025019   0.202728   0.736892   0.145236  0.0201529
  0.554264 0.00526775   0.204437   0.757662   0.147651  0.0203526
  0.744399  0.0116727   0.209749   0.749848   0.154412   0.020749
  0.539785  0.0100477   0.203933   0.687436   0.146852  0.0214487
  0.469092 0.00663163   0.200786   0.730525   0.141592  0.0209133
  0.366766 0.00237802   0.199084    0.72082   0.138091  0.0211555
  0.515581 0.00581557   0.202915   0.746435   0.144391  0.0205053
  0.569682 0.00305165   0.202605   0.774164   0.146171  0.0201569
  0.649801 0.00428778    0.20558   0.772032   0.149244  0.0201611
  0.517648 0.00331765   0.202367   0.751162   0.143916  0.0202778
------- 95 Percent Confidence Intervals -------
Rates | Standard Deviation
0.5664   |   0.122234
0.00621222   |   0.00392132
0.204361   |   0.00393705
0.749451   |   0.0350978
0.14672   |   0.00645022
Confidence Intervals for Each Rate:
Theta0: [0.518485,0.614316]
Theta1: [0.00467506,0.00774938]
Theta2: [0.202818,0.205905]
Theta3: [0.735692,0.763209]
Theta4: [0.144191,0.149248]
-----------------------------------------------
[illegible]
```

Supplementary Figure 8 BioNetGMMFit Parameter Estimates: Generated by the C++ executable, the "All Run Estimates" contains the labels of the model parameters with each row being a parameter estimate and its associated GMM cost.

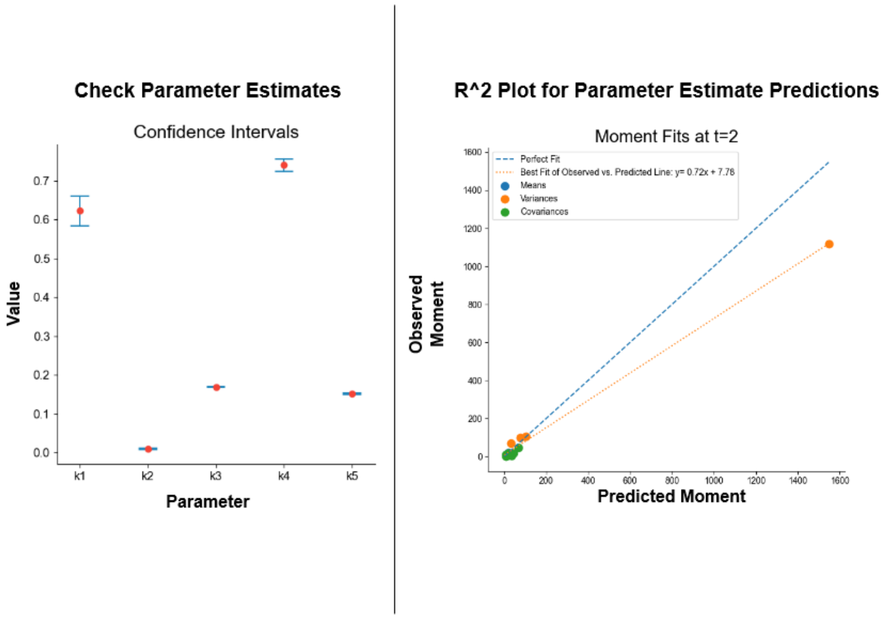

Supplementary Figure 9 BioNetGMMFit Graphical Outputs

Data Parameters	CD8+ T Cell Model	6 Protein Time Points Set A	6 Protein Time Points Set B	6 Protein Time Points Set C	6 Protein Time Set A Extreme
# of single cells used for estimation	719	5,000	5,000	5,000	5,000
# of single cells observed	653	5,000	5,000	5,000	5,000
Time Steps	1.0, 2.0	0.0, 0.5, 2.0	0.0, 0.5, 2.0, 7.0	0.0, 0.5, 2.0, 10.0, 20.0, 30.0	0.0, 0.5, 2.0

Supplementary Figure 10 Datasets Used For Estimation: The first row contain the labels of the model as well as its respective PSO hyperparameters and dataset contents used in BioNetGMMFit. Only two datasets are benchmarked, the 4 Protein CD8+ T cell and the simulated nonlinear Vav1 activation dataset. Each letter ”A”, ”B”, and ”C” in the ”6 Protein Time Points Set” columns indicate one unique set of time points of snapshot data used in the parameter estimation for the simulated 6 protein model. The ”Extreme” term in the last column represents a ten-fold multiplicative increase of particles and steps in PSO. For instance ”6 Protein Time Points Set A Extreme” is the PSO hyperparameter configuration that improves the parameter estimates from ”6 Protein Time Points Set A” as shown in Figure 10. Figure 11 provides more information regarding the PSO hyperparameters used for parameter estimation. The following row is the number of single cells in the initial conditions (i.e first time point) that are used for simulation by the BioNetGen model for estimation. The next row is the number of cells observed in the following time points. Cell counts remained constant across all time points in the simulated 6 protein Vav1 activation dataset.

Hyperparameter	CD8+ T cell Model	6 Protein Time Points Set A	6 Protein Time Points Set B	6 Protein Time Points Set C	6 Protein Time Set A Extreme
**Model Parameters**	**5**	**6**	**6**	**6**	**6**
**Particles**	**150**	**150**	**150**	**150**	**1,500**
**Steps**	**25**	**25**	**25**	**25**	**150**

Supplementary Figure 11 PSO and Model Parameters: Again, the first row indicates the model as well as its respective PSO hyperparameters and dataset contents. In this case, only two BioNetGen models were used, the 4 Protein CD8+ T cell model and the simulated 6 protein Vav1 activation model. The "Extreme" term in the last column represents a ten-fold multiplicative increase of particles and steps in PSO. For instance "6 Protein Time Points Set A Extreme" is the PSO hyperparameter configuration that improves the parameter estimates from "6 Protein Time Points Set A" as shown in Figure 10.

Number of Runs	CD8+ T cell Model (s)	6 Protein Time Points Set A (s)	6 Protein Time Points Set B (s)	6 Protein Time Points Set C (s)	6 Protein Time Set A Extreme (s)
**1**	**6**	**230**	**455**	**1,165**	**10,689**
**5**	**32**	**1,152**	**2,273**	**5,827**	**53,445**
**30**	**190**	**6,911**	**13,635**	**34,959**	**320,670**

Supplementary Figure 12 Run Times for Different Model Configurations: The 6 Protein Time Points Set A, B, and C columns show the change in run time with respect to the change in time steps used in Figure 10. 6 Protein Extreme provides some insight into how long the run time of an extreme number of particles and steps used in PSO. One run is considered to be one PSO estimate. More than one PSO estimate is required for confidence intervals.
