# Supplementary material for: BioNetGMMFit: estimating parameters of a BioNetGen model from time-stamped snapshots of single cells
Source: NPJ Syst Biol Appl. 2023 Sep 22;9:46. doi: 10.1038/s41540-023-00299-0 (PMC10516955; doi:10.1038/s41540-023-00299-0)
Supplement: Supplementary file 2 — Reporting Summary [file 41540_2023_299_MOESM2_ESM.pdf]

nature portfolio

Corresponding author(s): John Wu

Last updated by author(s): Apr 28, 2023

Reporting Summary

Nature Portfolio wishes to improve the reproducibility of the work that we publish. This form provides structure for consistency and transparency in reporting. For further information on Nature Portfolio policies, see our Editorial Policies and the Editorial Policy Checklist.

Statistics

For all statistical analyses, confirm that the following items are present in the figure legend, table legend, main text, or Methods section.

n/a	Confirmed	
☐	☒	The exact sample size (*n*) for each experimental group/condition, given as a discrete number and unit of measurement
☒	☐	A statement on whether measurements were taken from distinct samples or whether the same sample was measured repeatedly
☒	☐	The statistical test(s) used AND whether they are one- or two-sided<br>*Only common tests should be described solely by name; describe more complex techniques in the Methods section.*
☒	☐	A description of all covariates tested
☒	☐	A description of any assumptions or corrections, such as tests of normality and adjustment for multiple comparisons
☒	☐	A full description of the statistical parameters including central tendency (e.g. means) or other basic estimates (e.g. regression coefficient) AND variation (e.g. standard deviation) or associated estimates of uncertainty (e.g. confidence intervals)
☒	☐	For null hypothesis testing, the test statistic (e.g. *F*, *t*, *r*) with confidence intervals, effect sizes, degrees of freedom and *P* value noted<br>*Give P values as exact values whenever suitable.*
☒	☐	For Bayesian analysis, information on the choice of priors and Markov chain Monte Carlo settings
☒	☐	For hierarchical and complex designs, identification of the appropriate level for tests and full reporting of outcomes
☒	☐	Estimates of effect sizes (e.g. Cohen's *d*, Pearson's *r*), indicating how they were calculated

Our web collection on statistics for biologists contains articles on many of the points above.

Software and code

Policy information about availability of computer code

Data collection	We have three locations of application availability. We share our software on docker https://hub.docker.com/repository/docker/jhnwu3/bngmm/general, full Zip Build Folder https://zenodo.org/record/7733865, and a Web Demo https://bngmm.nchigm.org/. We also share our main GitHub repo with all of its available documentation https://github.com/jhnwu3/BioNetGMMFit.
Data analysis	All data analysis and collection was performed with the above software in Data Collection: https://github.com/jhnwu3/BioNetGMMFit. The dataset(s) supporting the conclusions of this article is(are) available in the BioNetGMMFit/example repository, [https://github.com/jhnwu3/BioNetGMMFit/tree/main/example]. For the full original CD8+ T Cell Experimental Dataset, see (https://dpeerlab.github.io/dpeerlab-website/dremi-data.html).

For manuscripts utilizing custom algorithms or software that are central to the research but not yet described in published literature, software must be made available to editors and reviewers. We strongly encourage code deposition in a community repository (e.g. GitHub). See the Nature Portfolio guidelines for submitting code & software for further information.

Data

Policy information about availability of data

All manuscripts must include a data availability statement. This statement should provide the following information, where applicable:

- Accession codes, unique identifiers, or web links for publicly available datasets
- A description of any restrictions on data availability
- For clinical datasets or third party data, please ensure that the statement adheres to our policy

he dataset(s) supporting the conclusions of this article is(are) available in the BioNetGMMFit/example repository, [https://github.com/jhnwu3/BioNetGMMFit/tree/main/example]. For the full original CD8+ T Cell Experimental Dataset, see (https://dpeerlab.github.io/dpeerlab-website/dremi-data.html).

Research involving human participants, their data, or biological material

Policy information about studies with human participants or human data. See also policy information about sex, gender (identity/presentation), and sexual orientation and race, ethnicity and racism.

Reporting on sex and gender	N/A
Reporting on race, ethnicity, or other socially relevant groupings	N/A
Population characteristics	N/A
Recruitment	N/A
Ethics oversight	N/A

Note that full information on the approval of the study protocol must also be provided in the manuscript.

Field-specific reporting

Please select the one below that is the best fit for your research. If you are not sure, read the appropriate sections before making your selection.

☒ Life sciences ☐ Behavioural & social sciences ☐ Ecological, evolutionary & environmental sciences

For a reference copy of the document with all sections, see nature.com/documents/nr-reporting-summary-flat.pdf

Life sciences study design

All studies must disclose on these points even when the disclosure is negative.

Sample size	Experimental dataset size was pre-determined. Simulated datasets were given sufficiently large numbers i.e 5,000 and 10,000 such that it would be sufficient for our software and to show that our software can accommodate large cell sizes.
Data exclusions	No data was excluded
Replication	Our manuscript is of a completely computational subject. All items can be replicated by seeing the code availability section. Please read the manuscript for further information.
Randomization	Again, our manuscript is of a completely computational subject. We randomly sample initial conditions from a lognormal distribution for simulated data.
Blinding	Computational manuscript, so no blinding required.

Reporting for specific materials, systems and methods

We require information from authors about some types of materials, experimental systems and methods used in many studies. Here, indicate whether each material, system or method listed is relevant to your study. If you are not sure if a list item applies to your research, read the appropriate section before selecting a response.

Materials & experimental systems

n/a	Involved in the study
☒	☐ Antibodies
☒	☐ Eukaryotic cell lines
☒	☐ Palaeontology and archaeology
☒	☐ Animals and other organisms
☒	☐ Clinical data
☒	☐ Dual use research of concern
☒	☐ Plants

Methods

n/a	Involved in the study
☒	☐ ChIP-seq
☒	☐ Flow cytometry
☒	☐ MRI-based neuroimaging
